# Minocycline-loaded PLGA electrospun membrane prevents alveolar bone loss in experimental peridontitis

**DOI:** 10.1080/10717544.2019.1709921

**Published:** 2020-01-08

**Authors:** Yihui Ma, Jinlin Song, Huthayfa N. S. Almassri, Dan Zhang, Ting Zhang, Yuting Cheng, Xiaohong Wu

**Affiliations:** a Stomatological Hospital of Chongqing Medical University, Yubei District, Chongqing, China;; b Chongqing Key Laboratory of Oral Diseases and Biomedical Sciences, Chongqing, China;; c Chongqing Municipal Key Laboratory of Oral Biomedical Engineering of Higher Education, Chongqing, China

**Keywords:** Minocycline, electrospinning, sustained release, periodontitis, osteogenesis

## Abstract

Minocycline (MINO) is a tetracycline antibiotic effective against most of the bacteria microorganisms related to periodontal disease. Additionally, MINO promotes bone *in vitro* and *in vivo*. The objective of the present study was to establish the protocol for the preparation of MINO-loaded poly (lactic-*co*-glycolic acid) (MINO-PLGA) electrospun membranes and to evaluate their effect on osteogenesis *in vitro* and in a rat model of periodontitis. The characterization of MINO-PLGA electrospun membranes was assessed by scanning electron microscopy, laser scanning confocal microscopy, and contact angle measurement. The drug release study showed a sustained diffusion of MINO from electrospun membranes over a period of 40 d. The MINO-PLGA membranes containing 2% of the drug exhibited better support of osteoblast proliferation and adhesion and was subsequently used *in vivo* in an experimental periodontitis model. Its therapeutic potential was evaluated by the measurement of alveolar bone loss (ABL), bone volume analysis, histological analysis, and immunohistochemistry. MINO-PLGA membrane increased alveolar crest height in the periodontitis model, inhibited the expression of the ligand of the receptor activator for nuclear factor-κB (RANKL), and promoted the expression of its inhibitor, osteoprotegerin. The study demonstrated that MINO-PLGA electrospun membranes may be applied to stimulate bone regeneration in periodontitis.

## 1. Introduction

Chronic periodontitis is one of the most common oral diseases. The continuous alveolar bone loss (ABL) caused by this condition leads to teeth loosening and, eventually, teeth loss (Pihlstrom et al., 2005).

Minocycline (MINO) is a broad-spectrum tetracycline antibiotic. It is one of the most frequently used antibiotics in fighting most of the bacteria responsible for periodontitis (Oliveira et al., 2006). In addition to its antimicrobial activity, MINO exhibits additional effects on periodontal diseases (Vernillo & Rifkin, 1998). MINO has been shown to inhibit bone resorption and promote bone formation. Pedro and coworkers documented that this antibiotic at 1 mg/ml, i.e. the standard therapeutic concentration in plasma, significantly increased the proliferation of human bone marrow osteoblastic cells without affecting their phenotype and function (Bettany & Wolowacz, 1998). Tsuneyasu and collaborators demonstrated that MINO inhibited osteoclast-like cell formation by mouse bone marrow cells (BMCs) induced by 1α, 25-dihydroxyvitamin D_3_ (1α, 25(OH)_2_D_3_) (Bettany et al., 2000). However, a high concentration of an antibiotic in the periodontal pockets may directly or indirectly affect the viable cells of the supportive periodontal tissue, in particular, the bone-forming osteoblasts (Gomes & Fernandes, 2007; Almazin et al., 2009). Moreover, high levels of MINO can produce dose-dependent toxic effects on cells, inhibiting the proliferation and differentiation of osteoclasts (Bettany & Wolowacz, 1998). To avoid high local concentration of antibiotics, the effectiveness of sub-antimicrobial doses of MINO and its local delivery became the focus of recent studies (Babich & Tipton, 2002). It has been documented that the minimal inhibitory concentration (MIC) of MINO against Treponeme is less than 0.125 μg/mL (Okamoto-Shibayama et al., 2017) and against Actinobacillus actinomycetemcomitans (Aa) is 0.25 μg/mL (Takahashi et al., 2006). The study by Maria and colleagues showed that the MIC of tetracyclines was 0.25 μg/mL for Porphyromonas gingivalis and Prevotella intermedia, and ≤0.125 μg/mL for *Prevotella nigrescens* (Andrés et al., 1998). The application of local drug delivery systems to provide a sustained release of antimicrobial agents in the periodontal pockets for prolonged time has also been investigated (Tonetti et al., 1990; Kornman, 1993; Roskos et al., 1995). Specifically, further studies on the sustained release of MINO are required to achieve osseointegration and ensure an effective antibacterial concentration.

The electrospinning technique allows for obtaining several types of scaffolds with various characteristics by applying electrostatic principles. Electrospinning used in drug delivery (Chakraborty et al., 2009; Hu et al., 2014) and tissue regeneration (Bonino et al., 2012; Zhao et al., 2013) can generate substrates with attractive features such as the similarity to the extracellular matrix (ECM), high loading capacity, high encapsulation efficiency, ease of use, and cost-effectiveness. Poly (lactic-*co*-glycolic acid) (PLGA) is a promising biodegradable material for local release systems that allow sustained delivery of high concentrations of drugs at the site of application (Kumbar et al., 2008; Chen et al., 2012). Importantly, PLGA has been approved by the Food and Drug Administration (FDA) because it is nontoxic, does not activate inflammatory reactions, and is easily biodegradable (Ali et al., 1993). A critical advantage of PLGA is that it offers the possibility of controlling the rate of drug delivery by changing the ratio between the lactic and glycolic acid monomers (Ranganath & Wang, 2008). The objective of the present study was to develop the methodology of preparation of MINO-loaded PLGA electrospun membranes, and to analyze their properties, such as the morphology, surface wettability, drug release, drug degradation, and interaction with osteoblasts. Additionally, in vivo experiments were performed to determine whether MINO-PLGA electrospun membranes can promote alveolar bone augmentation in ligature-induced experimental periodontitis.

## 2. Materials and methods

### 2.1. Materials

Poly (d,l-lactide-*co*-glycolide) (LA:GA = 50:50, *M*_W_ = 70,000) was supplied by Jinan Daigang Co. (Jinan, China). Minocycline (MINO) was purchased from Sigma-Aldrich (St Louis, MO), and the MINO ointment (Periocline^®^) from Sunstar Inc. (Osaka, Japan). The osteoblasts were cultured in alpha-modification of Eagle’s medium (α-MEM) (Hyclone, Logan, UT) supplemented with 10% fetal bovine serum (FBS) (Hyclone, Logan, UT) and 1% penicillin–streptomycin–amphotericin (Hyclone, Logan, UT). All other chemicals used in the study were purchased from Mengbio Regents Company (Chongqing, China).

### 2.2. Synthesis of PLGA membranes loaded with MINO

The polymer solution was prepared by dissolving 0.7 g PLGA in 2 ml chloroform. To ensure complete dissolution, the mixture was stirred at room temperature for 1 h. Next, different concentrations of MINO powder (1, 2, and 3 wt%, relative to the polymer weight) were added into the PLGA solution and stirred for an additional 1 h. The resulting solution was poured into a 5 mL syringe (5 mL) with a metal needle. A voltage of 15 kV was applied to the syringe needle and the collector was placed 18 cm from the needle tip. The solution flow rate was set at 3 mL/h. The electrospinning was carried out at room temperature (22 ± 2 °C) and a relative humidity of 50 ± 5%. The collected fiber membranes were kept overnight in a desiccator to ensure complete evaporation of the solvent.

### 2.3. Characterization of MINO-loaded PLGA membranes

#### 2.3.1. Scanning electron microscopy (SEM)

The morphology of MINO-PLGA electrospun membranes was examined by scanning electron microscope (SEM, S-3000N, JEOL, Tokyo, Japan) at an accelerating voltage of 20 kV. First, the fiber membranes were carefully fixed on stubs, then gold coated by a sputtering device for 60 s before SEM observation. The average diameters were calculated using the Image J software (National Institutes of Health, Bethesda, MD) (50 randomly selected fibers per sample).

#### 2.3.2. Fourier transforms infrared spectroscopy (FTIR)

FTIR (Thermo Scientific Nicolet iS5, Waltham, MA) was carried out to determine the chemical composition of MINO powder, PLGA and 2% MINO-PLGA electrospun membranes. The samples were observed by attenuated total reflection (ATR). The wave number range of infrared absorption was 500–4000 cm^−1^.

#### 2.3.3. Laser scanning confocal microscopy

The characteristic yellow-green fluorescence of MINO can be observed under certain conditions (Dodiuk-Gad et al., 2006). Therefore, specimens of electrospun fibers were placed on a glass slide and observed under a laser-scanning confocal microscope (TCS SP8X, Leica, Wetzlar, Germany) using the excitation wavelength of 375 nm.

#### 2.3.4. Contact angle (CA) measurement

The surface wettability of electrospun membranes was determined by measuring the static contact angle using a video contact angle instrument (Siber Hegner, Shanghai, China). Specimens, sized 1 × 1 cm, were collected, placed on the testing plate, and covered with a drop of distilled water (the average drop contained about 0.05 mL of solution). Readings were taken within the first 10 s after the deployment of the drop. Every group had 5 samples, and the contact angle of each sample was calculated by determining at three different locations. The means and standard deviations were shown as the results.

### 2.4. *In vitro* drug release

The release of MINO from electrospun membranes was determined in vitro by cutting the membranes into 2 × 2 cm pieces, recording their weight, and incubating at 37 °C in 4 mL of phosphate-buffered saline (PBS). The total volume of PBS was collected at different time points and the amount of MINO released from the membrane was measured by absorbance at 375 nm (Nanodrop2000, Thermo Fisher Scientific, Waltham, MA), and the optical density (OD) values were converted to concentrations using a standard curve. The soaking of MINO-loaded electrospun membranes was continued in a fresh 4 mL aliquot of PBS. The results were expressed as the cumulative amount of released MINO at each time point, according to the formula:

$$\text{Cumulative}\;\text{amount}\;\text{of}\;\text{release}\;\left(\%\right)={\text{M}}_{t}/{\text{M}}_{a}\times \text{1}00\%,$$

in which M_t_ is the total amount of MINO released from the membrane at time t, and M_a_ is the total amount of MINO in the membrane.

To describe the kinetics and mechanism of MINO release from electrospun membranes, the release data were fitted in different mathematical models, including the zero-order, first-order, Higuchi, and Ritger–Peppas models to calculate correlation coefficients, *R*^2^, and release rate constants, K (Costa & Sousa Lobo, 2001).

### 2.5. *In vitro* degradation of membranes

The membranes were immersed in PBS and incubated at 37 °C for different periods of time. Subsequently, the membranes were washed with distilled water, dried, and weighed. The degradation rate was calculated using the following equation:

$$\text{Weight}\;\text{loss}\;\left(\%\right)={\left({\text{W}}_{0}-{\text{W}}_{t}\right)}_{}/{\text{W}}_{0}\times \text{1}00\%,$$

in which W_0_ is the initial weight of the membrane, and W_t_ is the weight at time t. Each experiment was done in triplicate.

### 2.6. Cell culture

Newborn Sprague–Dawley rats were soaked in 75% alcohol for 10 min, and cranial bones were collected and cultured for several days to obtain osteoblast cells. The osteoblasts were cultured in α-MEM supplemented with 10% FBS and 1% penicillin–streptomycin–amphotericin in 5% CO_2_ at 37 °C. The medium was replaced every 2–3 days.

#### 2.6.1. Cell proliferation

Osteoblasts were seeded in a 24-well plate at a density of 2 × 10^4^ cells/well. After 24 h, the cells were incubated at 37 °C with PLGA membranes containing 1, 2, and 3 wt% PLGA-MINO. Cultures without membranes served as controls. After 1, 3, and 7 days, cell proliferation was determined by the CCK-8 assay (Tongren, Japan) according to the manufacturer’s instructions. The OD values were measured using an ELISA plate reader at 375 nm (Bio-Tek, Winooski, VT).

#### 2.6.2. Cell attachment

After culturing for 3 days, the cells were washed three times with PBS, fixed in 2.5% paraformaldehyde for 2 h at 4 °C, and dehydrated in a series of ethanol washes (30%, 50%, 70%, 80%, 90%, 95%, and 100%). The cells were then vacuum-dried at low temperature for 24 h and observed by SEM.

### 2.7. Animals and experimental design for the induction of periodontitis

After a 1-week acclimation period, female Sprague–Dawley rats (7 weeks old) from the Institute of Experimental Animal Center of Chongqing Medical University were assigned randomly to five groups, with 5 rats in each group: (1) Control (sham), (2) ligation, (3) ligation + PLGA, (4) ligation + 2% MINO-PLGA, (5) ligation + Periocline^®^. All experimental procedures were approved by the Ethics Committee of the Affiliated Stomatological Hospital of Chongqing Medical University (Approval no. (2019) 34). Under anesthesia induced by intraperitoneal injection of 4 ml/kg of 10% chloral hydrate, an orthodontic steel wire (0.2 mm in diameter) was ligated around the cervix of the mandibular first molars for 4 weeks to induce experimental periodontitis for 4 weeks. This procedure was not performed in the control group. During this period, the rats were examined once a week to ensure the wire did not loosen or fall off, and to replace it, if necessary. After the periodontitis model was established, the ligatures were removed, and the periodontal pockets were left without treatment (control group), or a PLGA membrane (ligation + PLGA group) and a 2% MINO-PLGA membrane (ligation + MINO-PLGA group) were inserted into the periodontal pockets immediately after the removal of the ligature, or Periocline^®^ was injected into the periodontal pockets once a week (3 weeks and 6 weeks, *n* = 5 at each time point).

#### 2.8.2. Micro-computed tomography (micro-CT) and bone analysis

At 3 and 6 weeks after the removal of the ligatures, rats were sacrificed by an overdose of chloral hydrate. The maxillae were fixed in 4% paraformaldehyde and analyzed by micro-CT (Viva CT40, SCANCO Medical, Brüttisellen, Switzerland) at a resolution of 15 mm, the energy of 70 kV, and 114 mA. The alveolar bone loss (ABL) was measured in two-dimensional (2 D) micro-CT sections. The linear distance of ABL was measured from the cementoenamel junction (CEJ) to the alveolar bone crest (ABC) at the distal and mesial root of the upper first molar (Hu et al., 2014). The bone volume/tissue volume (BV/TV) parameter was estimated in a three-dimensional region of interest (ROI) using the SCANCO analysis software. The ROI included axially a rectangle with length and width encompassing the entire crown, and vertically a height from the CEJ to the apex. The first molars were then removed and the remaining bone volume in the ROI has been measured.

#### 2.8.3. Histological analysis of alveolar bone

After micro-CT imaging, the maxilla bones were decalcified in 10% EDTA at 37 °C for 1 month. Subsequently, the samples were dehydrated, embedded in paraffin, and sliced into 5 μm thick sections in the mesiodistal direction. The sections were placed on glass slides, dried at a high temperature and stained by hematoxylin and eosin (H&E) (Solabao, Beijing, China).

#### 2.8.4. Immunohistochemistry (IHC)

The expression of the osteogenic marker osteogenic growth peptide (OPG) and the osteoclastic marker RANK ligand (RANKL) was evaluated at 3 and 6 weeks after ligation. Image analysis was performed using Image J (NIH Image J system, Bethesda, MD).

### 2.9. Statistical analyses

The data are presented as the mean ± standard deviation (SD). One-way analysis of variance (ANOVA) and the Student–Newman–Keuls (SNK-q) test were employed to determine statistical significance. All calculations were performed using the SPSS software (SPSS, Chicago, IL), version 17.0. *p* < .05 was considered statistically significant.

## 3. Results

### 3.1. Characterization and hydrophilicity of MINO-loaded PLGA membranes

**Figure 1.**
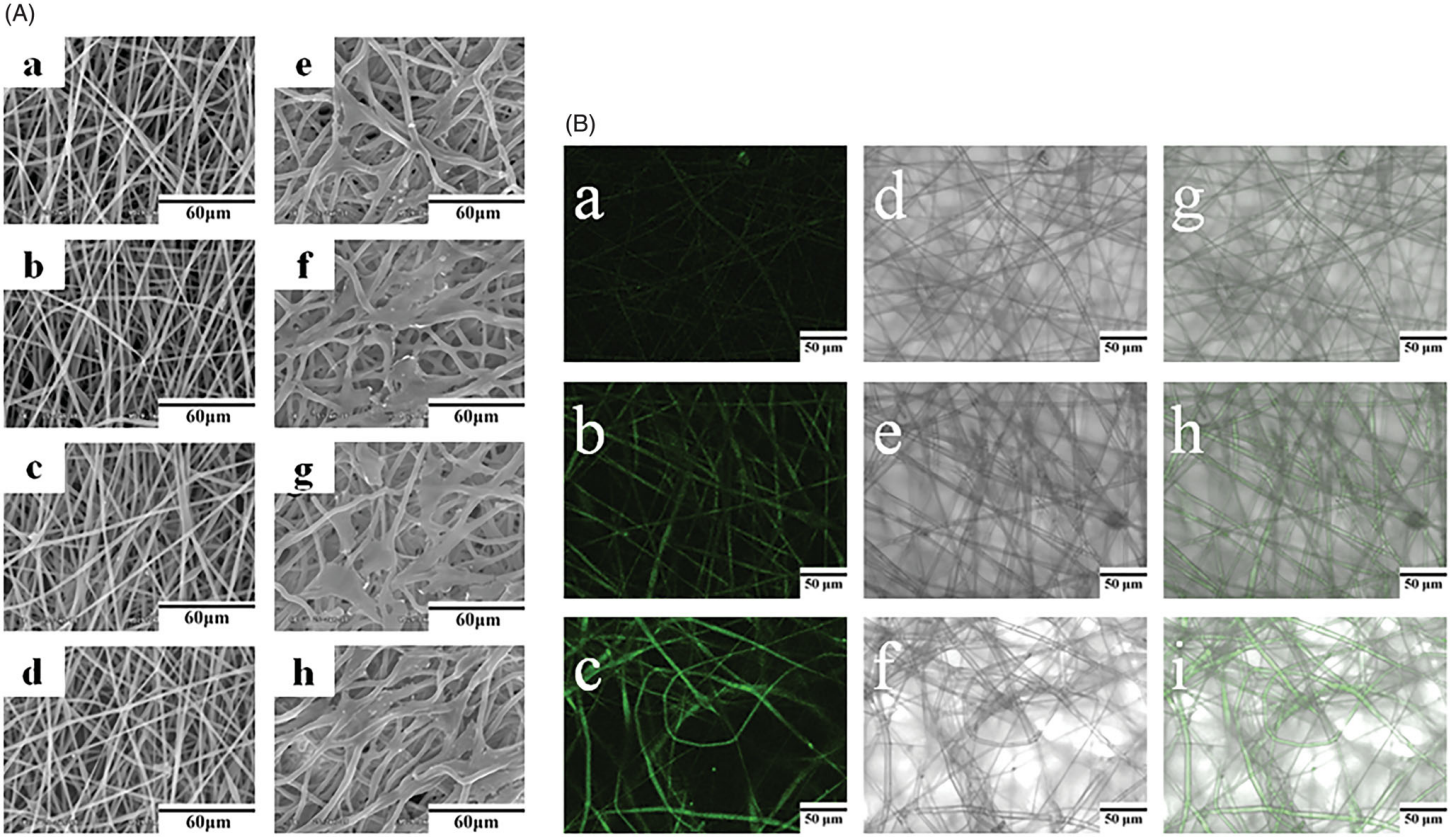
(A) SEM images of fibers and cells grown on the membranes (SEM scale bar = 60 µm) of PLGA (a, e), 1% MINO-PLGA (b. f), 2% MINO-PLGA (c, g), and 3% MINO-PLGA (d, h), (B) laser scanning confocal microscopy images of 1% MINO-PLGA (a, d, g), 2% MINO-PLGA (b, e, h), 3% MINO-PLGA (c, f, i). Fluorescent picture, bright field picture and composite picture from left to right respectively (scale bar = 50µm).

The morphology of PLGA fibers containing 0%, 1%, 2%, and 3% of MINO observed is demonstrated in Figure 1(A) by SEM. The fibers were typically ribbon-shaped and did not contain faults such as beads or broken strands. The presence of varying concentrations of MINO in PLGA fibers did not affect their morphology. Table 1 documents that the average diameters of PLGA fibers containing 0%, 1%, 2%, and 3% of MINO were 1661, 1618, 1579, and 1569 nm, respectively. Although the diameters of PLGA fibers tended to decrease with increasing MINO concentrations, the differences did not reach statistical significance (*p* > .05).

**Table 1.** The fiber diameters and contact angles of MINO-PLGA.

Group	Fiber diameter (μm)	Contact angle (deg)
0%MINO-PLGA	1.661 ± 0.325	86.970 ± 3.698
1%MINO-PLGA	1.618 ± 0.253	74.354 ± 6.917*
2%MINO-PLGA	1.579 ± 0.316	73.686 ± 9.642*
3%MINO-PLGA	1.569 ± 0.269	68.684 ± 12.580*

The data represented the mean ± SD, *n* = 3, *p < .05.

**Figure 2.**
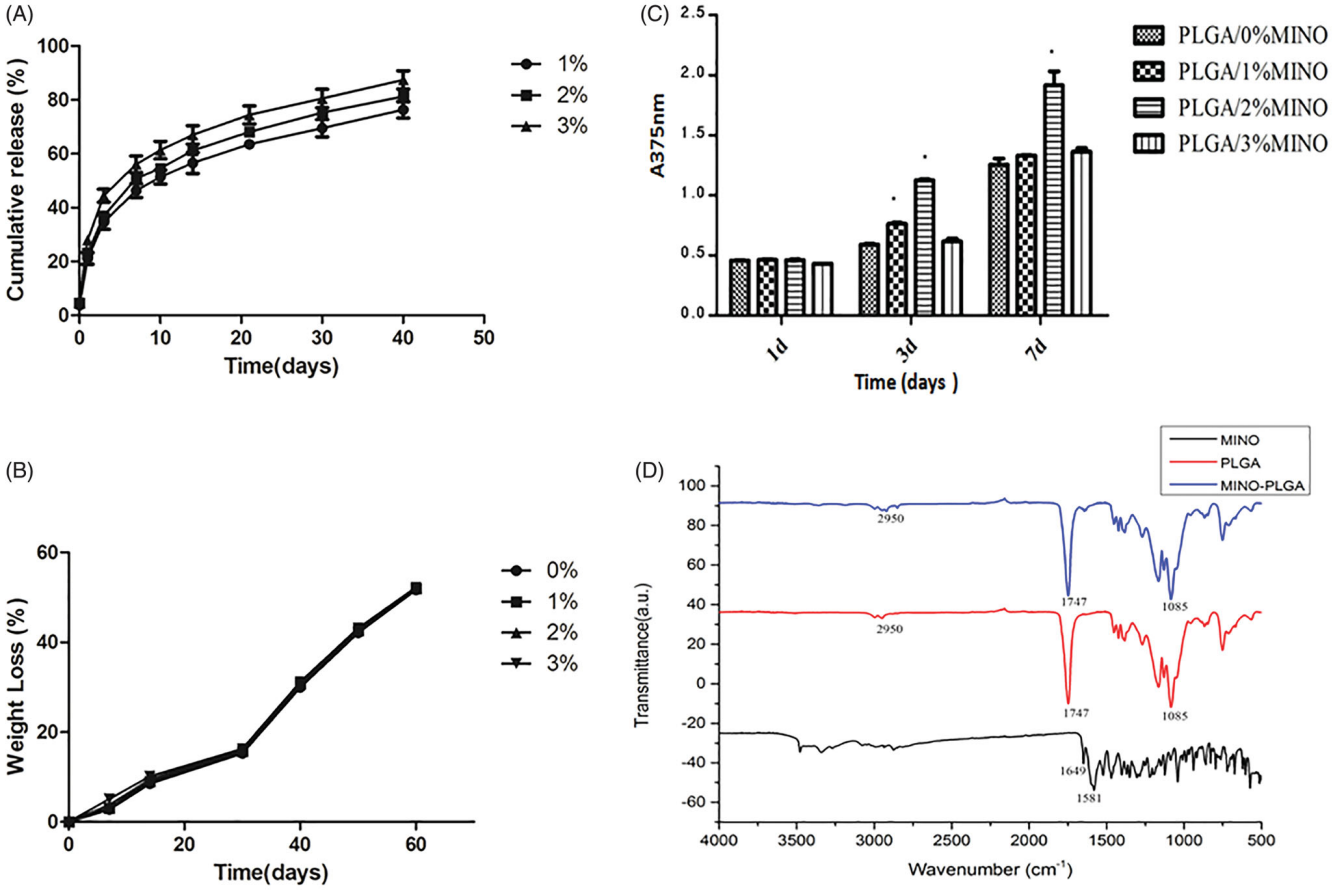
(A) MINO release properties from the 1%, 2%, and 3% MINO-PLGA membranes, (B) weight loss of 0%, 1%, 2%, and 3% MINO-PLGA membranes. The data represented the mean ± standard deviation (*n* = 7 for release and *n* = 3 for degradation), (C) CCK-8 assays of cells cultured on 0%, 1%, 2%, and 3% MINO-PLGA membranes. The data represented the mean ± SD, *n* = 3, *p < .05, (D) FTIR spectra of MINO, PLGA and 2% MINO-PLGA electrospun membranes.

The FTIR spectra of MINO, PLGA, and 2% MINO-loaded PLGA electrospun membranes are presented in Figure 2(D). MINO showed multiple complex absorption peaks in the range of 500–1750 cm^−1^, because the molecular structure contains four benzene rings. MINO had characteristic IR peaks at 1649 (amide band I) and 1581 (amide band II). The PLGA spectrum showed the bands of the –CH, –CH2, –CH3 stretching vibrations (2950 cm^−1^), the carbonyl C = O stretching vibrations (1 747 cm^−1^), and the C–O stretching vibrations (1085 cm^−1^). All characteristic peaks for MINO and PLGA could be observed in the FTIR spectrum of MINO-PLGA electrospun membranes.

Laser scanning confocal microscopy (Figure 1(B)) was conducted to visualize the presence and distribution of MINO in nanofiber membranes. Fibers containing 1%, 2%, and 3% MINO exhibited string-like yellow-green signals, indicating a relatively homogeneous MINO distribution. The intensity of fluorescence intensity was enhanced with the increase in MINO loading.

The results of CA measurements for stable water droplets placed on the nanofiber mats are listed in Table 1. The final CA values for PLGA nanofibers loaded with 0%, 1%, 2%, and 3% of MINO were 86.970° ± 3.698°, 74.354° ± 6.917°, 73.686° ± 9.642°, and 68.684° ± 12.580°, respectively. The addition of MINO clearly raised the hydrophilicity of the nanofibers (*p* < .05). As the concentration of MINO increased, the CA decreased but the changes were not statistically significant (*p* > .05).

### 3.2. *In vitro* release and degradation of MINO-PLGA

The kinetics of MINO release from PLGA fibers are illustrated in Figure 2(A). An initial burst of release was observed on the first day, with 21.11%, 23.17%, and 28.06% of the antibiotic freed from 1%, 2%, and 3% MINO-PLGA, respectively. Subsequently, a relatively fast release was noted until day 7. A slow but sustained release continued for 40 d, with 76.33%, 81.26%, and 87.42% of MINO released at that time from 1%, 2%, and 3% MINO-PLGA, respectively. Of note, the amount of MINO released from the electrospun membranes increased with higher concentrations (*p* < .05). Table 2 presents the fitting of different models to the kinetic data, together with the corresponding *R*^2^ values. The best fit was obtained with the Ritger–Peppas equation, which showed the best correlation with the data on MINO release (*R*^2^>0.990).

**Table 2.** Release kinetic modeling for optimized batch of MINO-PLGA membrane.

Batch	Zero order	First order	Higuchi model	Rigter–Pettas model
1%MINO-PLGA	*R*^2^ = 0.745	*R*^2^ = 0.922	*R*^2^ = 0.954	*R*^2^ = 0.992
2%MINO-PLGA	*R*^2^ = 0.744	*R*^2^ = 0.920	*R*^2^ = 0.952	*R*^2^ = 0.990
3%MINO-PLGA	*R*^2^ = 0.705	*R*^2^ = 0.920	*R*^2^ = 0.952	*R*^2^ = 0.990

Additionally, the cumulative weight loss over time in the four groups of MINO-PLGA electrospun membranes was measured (Figure 2(B)). During the first 30 d, the membranes exhibited a very slow weight loss, indicating a low rate of degradation. The loss of weight was accelerated after this period, and at 60 d, the residual weight of 0%, 1%, 2%, and 3% MINO-PLGA was approximately 52%. The weight loss of the electrospun membranes did not correlate with MINO concentration (*p* > .05).

### 3.3. Cell attachment and proliferation

The morphology of osteoblasts cultured for 3 d on electrospun membranes is illustrated in Figure 1(A) by SEM. The cells were attached to the surface by numerous discrete filopodia and grew well on the membranes.

The proliferation of osteoblasts on the surface of membranes is shown in Figure 2(C). The number of cells increased with time in all groups. On day 1, there was no statistically significant difference among the four groups (*p* > .05). However, from day 3, significantly higher cell proliferation was detected in cultures grown on the MINO-PLGA membranes, in particular in the 2% MINO-PLGA group (*p* < .05), compared to cells cultured on the PLGA membranes. These findings suggest that MINO-PLGA nanofibers, especially those containing 2% MINO, potentiate osteoblast cell growth.

### 3.4. *In vivo* studies

#### 3.4.1. Micro-CT findings

**Figure 3.**
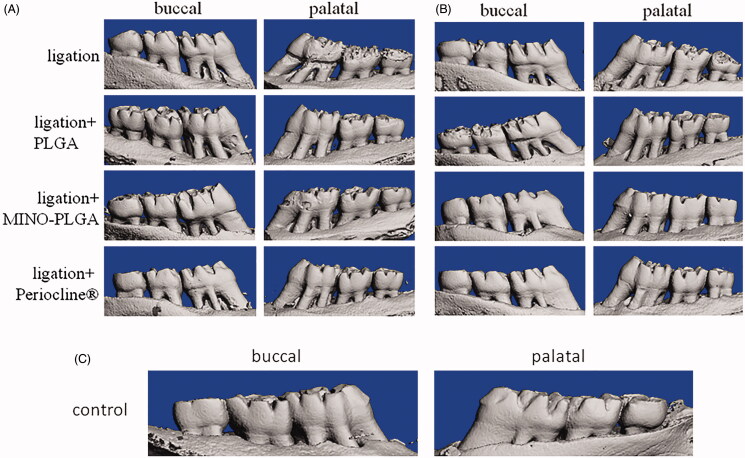
Reconstructed three-dimensional micro-CT images (buccal and palatal view) of the maxilla first molars alveolar bone level in control (C), ligation, ligation + PLGA, ligation + MINO-PLGA and ligation + Periocline® group at 3 weeks (A) and 6 weeks (B).

**Figure 4.**
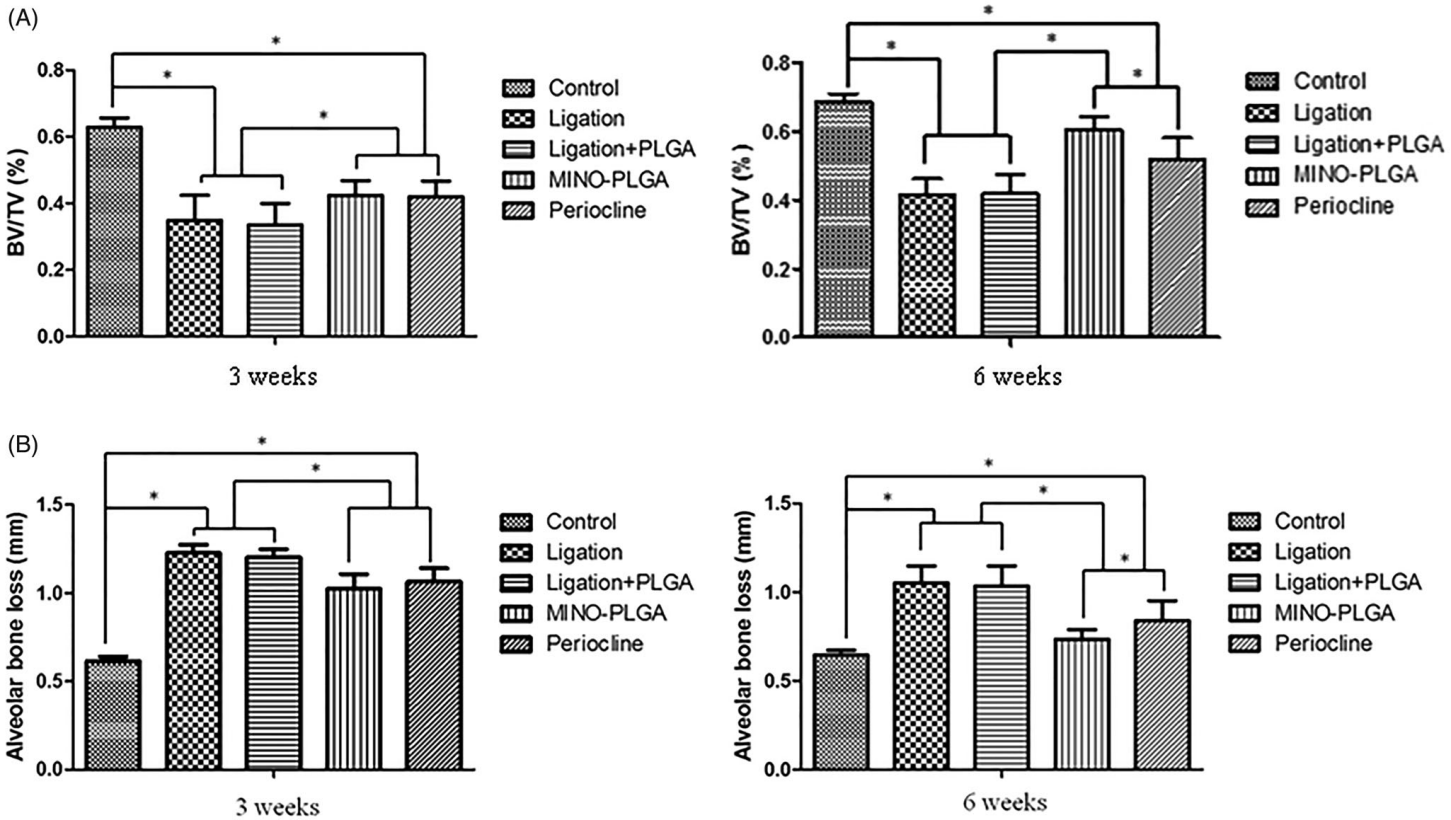
(A) The degree of alveolar bone loss was evaluated by measuring the CEJ-ABC distance at the distal and mesial root of the first molar from the reconstructed micro-CT images, (B) analysis of micro-CT volumetric parameters: bone volume/tissue volume (BV/TV). The data represented the mean ± SD, *n* = 5, *p < .05.

3D reconstruction by micro-CT revealed that in comparison with the ligation and ligation + PLGA groups, the reduction in alveolar crest height was partially prevented in the ligation + MINO-PLGA group and the ligation + Periocline^®^ group at 3 and 6 weeks (Figure 3). Moreover, the quantification of bone parameters demonstrated that MINO-PLGA and Periocline^®^ treatment significantly prevented the ligature-induced decreases in BV/TV when compared with the ligation and ligation + PLGA groups at 3 and 6 weeks (*p* < .05; Figure 4(A)). The alveolar bone loss (ABL) was assessed using the images of 2 D micro-CT sections (Figure 4(B)). The ABL values in MINO-PLGA, Periocline^®^, ligation, and PLGA groups were 1.02 ± 0.084 mm, 1.06 ± 0.076 mm, 1.23 ± 0.049 mm, and 1.20 ± 0.046 mm, respectively, at 3 weeks and 0.73 ± 0.055 mm, 0.84 ± 0.111 mm, 1.05 ± 0.097 mm, and 1.03 ± 0.114 mm at 6 weeks. The difference in ABL between MINO-PLGA and Periocline^®^ treatments were statistically significant at 6 weeks (*p* < .05).

#### 3.4.2. Histological analyses of alveolar bone

**Figure 5.**
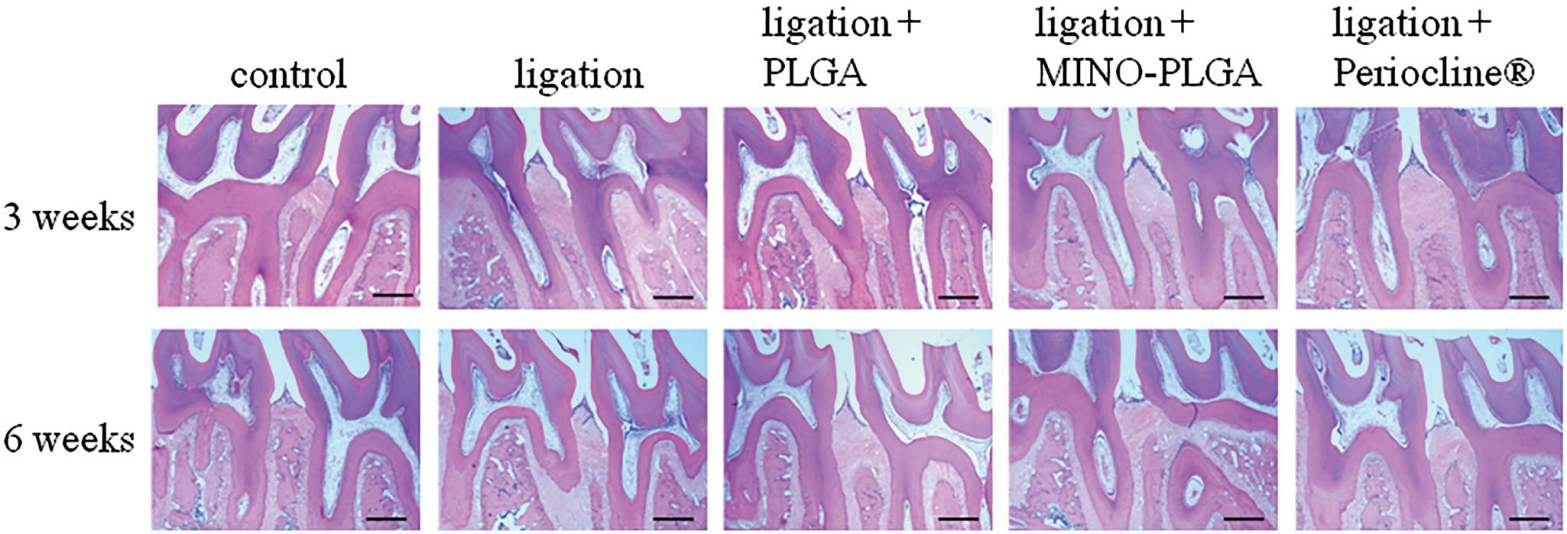
Histological observation (H&E stain) of maxillary periodontal tissue after 3 and 6 weeks (40 × magnification, bar = 500 µm).

Histologically, normal periodontal structure around the upper first molar was observed in the control group, while the ligation and ligation + PLGA groups exhibited the loss of alveolar bone and destruction of the integrity of periodontal tissue between the first and second upper molars consistent with periodontitis induction (Figure 5). In contrast, the height of the alveolar bone in the MINO-PLGA and Periocline^®^ groups increased and the periodontal structure was better preserved at 3 and 6 weeks.

#### 3.4.3. Expression of RANKL and OPG

**Figure 6.**
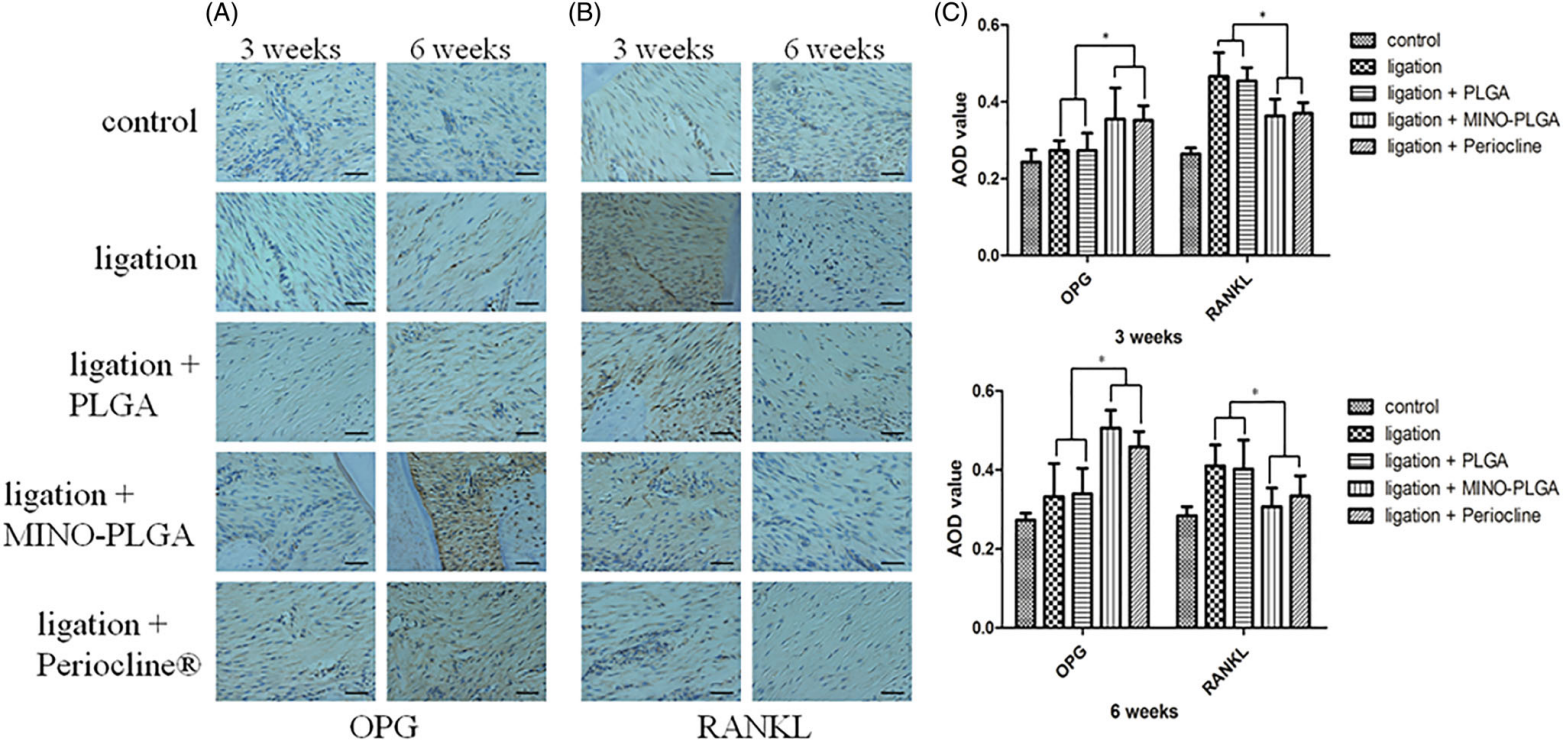
IHC staining and the corresponding quantitative analysis of OPG and RANKL after 3 and 6 weeks (400 × magnification, bar = 50 µm). The results represent the mean ± SD, *n* = 3, *p < .05.

The results of IHC staining for OPG and RANKL proteins are illustrated in Figure 6(A,B). In comparison with the ligature and ligation + PLGA groups, the expression of RANKL decreased, and OPG increased in the MINO-PLGA and Periocline^®^ groups at 3 and 6 weeks. This difference was also evident in the quantitative analysis of the level of expression of OPG and RANKL (Figure 6(C)). There was no statistically significant difference between MINO-PLGA and Periocline^®^ groups (*p* > .05).

## 4. Discussion

The inflammation caused by the oral pathogen and the resorption of alveolar bone resorption are essential features of periodontitis. Therefore, suppressing gingival tissue inflammation and preventing periodontal destruction may represent a strategy to ameliorate periodontal disease (Hatipoğlu et al., 2015). The main objective of the current study was to fabricate MINO-loaded electrospun PLGA membranes capable of a sustained release of the antibiotic to ensure its adequate concentration in the periodontal pockets, The MINO-PLGA membranes were expected to stimulate bone formation while retaining the antibacterial activity.

Several studies have demonstrated that a therapeutic level of MINO promotes the proliferation and differentiation of human bone marrow osteoblastic cells (Gomes & Fernandes, 2007), mouse bone marrow cells (Nagasawa et al., 2011), and osteoblasts cells (Kinugawa et al., 2012). Additionally, MINO inhibits the proliferation of bone marrow-derived macrophages (BMMs) (Kinugawa et al., 2012). The current work found that MINO-PLGA, and the membrane containing 2% MINO in particular, promote the proliferation of osteoblasts. This conclusion is based on the attachment of osteoblasts to the membrane and the results of the CCK-8 assay showing an increase in cell number.

In the *in vitro* release study, electrospun membranes released MINO in a biphasic mode with an initial burst release followed by a sustained release of the antibiotic until the end of the protocol. Moreover, the values of the diffusion coefficient (*n*) in the Ritger–Peppas equation were less than 0.5 for all groups, indicating the diffusion as the mechanism of drug release from the electrospun membranes (Costa & Sousa Lobo, 2001; Khan et al., 2017). Generally, the drug release mechanism and kinetics for biodegradable materials can be divided into three stages: (1) initial burst release; (2) diffusion-controlled release; and (3) degradation-controlled release (Hsu et al., 2016). During the electrospinning process, most molecules of the drug are dispersed within the PLGA matrix; however, some of them may be located on the fiber surface and be responsible for the initial burst release (Hsu et al., 2016; Janjic et al., 2017). After the initial burst, the pattern of drug release is controlled not only by the diffusion but also by the degradation of the matrix material (Hsu et al., 2016; Janjic et al., 2017). These processes resulted in the relatively constant release rate of MINO after the minor initial burst observed in this study.

Periocline^®^ is a bio-absorbable drug in the form of a sustained local drug delivery system, consisting of 20 mg/g minocycline hydrochloride in a matrix of hydroxyethyl-cellulose (Yang et al., 2018). The release rate of Periocline^®^ is dependent on the degradation of the matrix material, and antibacterial activity can last for approximately 7 d (Yang et al., 2018). Periocline^®^ was shown to increase the concentration of MINO in the periodontal pocket, but the rate of drug release is not constant (Vandekerckhove et al., 1998; Liu et al., 2013). Typically, 80–90% of MINO is released within 2–3 d from placing the compound in the periodontal pocket (Vandekerckhove et al., 1998; Liu et al., 2013), and then the drug concentration becomes significantly reduced. As a consequence, drug concentration in the periodontal pocket fluctuates greatly, which is not conducive to osteogenesis (Vandekerckhove et al., 1998; Liu et al., 2013). A commonly used method of administration of Periocline^®^ in clinical practice is to perform weekly injections in the periodontal pocket for one month. The requirement of repeated multiple visits may create some inconvenience for patients, leading to poor adherence to the treatment procedure.

The initial burst followed by a sustained slow release of MINO lasting for 40 d, demonstrated in the present work, enables the electrospun membranes to deliver a desired therapeutic concentration of the drug over an extended period. This property of MINO-PLGA promoted bone repair, raising the prospects for clinical applications. However, mixing MINO with the polymer and co-electrospinning could not prevent completely the burst release of the antibiotic (Chen et al., 2012). Therefore, a better method of loading the drug into the electrospun membranes will be explored in future research, with the objective of further reduction of the burst release.

A review by Piergiorgio and coworkers concluded that the degradation time of PLGA (MW =70,000) is 6–12 weeks (Baino, 2011; You et al., 2005a, 2005b; Gentile et al., 2014). Young and collaborators have found that the residual weight of the PGA/PLA (50/50) fibers after 45 d was approximately 40% of the initial weight (You et al., 2005a, 2005b). PLGA is hydrolyzed in a process involving de-esterification (Gentile et al., 2014), and the rate of degradation rate depends on multiple parameters, such as its molecular weight, crystallinity, morphological structure, and copolymer composition (Gentile et al., 2014). The PLGA monomers are removed through natural pathways after degradation. Typically, the co-polymers of PLGA offer the possibility of superior control of degradation by changing the ratio between its monomers. Therefore, PLGA preferred for drug delivery (You et al., 2005a, 2005b; Baino, 2011). MINO, in the concentration range used in the present study, did not affect the degradation rate of PLGA membranes. During the first 30 d, the PLGA membranes showed a very slow rate of weight loss; this interval is referred to as the induction period. The rate of weight loss was increased afterward, and at 60 d, the residual weight of PLGA was approximately about 52% of the original. Dario and colleagues suggested that the maintenance of fiber structure is essential for the sustained drug release at a relatively constant rate (Puppi et al., 2010).

The micro-CT results indicated a positive effect of MINO on alveolar bone formation, and this finding was confirmed by a marked decrease in ABL and an increase in the BV/TV value. There was no significant difference in ABL and bone volume between the ligation and ligation + PLGA groups, indicating that the PLGA electrospun membranes alone do not affect alveolar bone height and bone volume. The improved values of ABL and BV/TV in the MINO-PLGA group versus the Periocline^®^ group at 6 weeks may be attributed to the long-term sustained release of MINO from the membranes.

RANKL is essential for osteoclast differentiation. Conversely, OPG, a natural inhibitor of RANKL, inhibits this process. Osteoclast precursors express the receptor of RANKL (RANK), and the ligand-receptor interaction induces the precursors to differentiate into osteoclasts (Harada & Takahashi, 2011). In addition, osteoblasts express OPG that inhibits osteoclast proliferation and bone resorption (Lu et al., 2013). The changes in the RANKL-to-OPG ratio promote osteoclasts differentiation (Theoleyre et al., 2004; Lossdörfer et al., 2011). The presented results regarding the expression of RANKL and OPG indicate that MINO can increase the formation of osteoblasts formation by downregulating RANKL and upregulating OPG.

## 5. Conclusion

MINO-PLGA electrospun membranes were successfully prepared and demonstrated effective surface wettability and the ability to promote osteoblast attachment and proliferation *in vitro*. Despite an initial burst release of MINO, the MINO-PLGA electrospun membranes showed adequate rate and duration of sustained release in vitro and improved osteogenesis after periodontitis *in vivo*. The use of MINO-PLGA membranes can reduce the number of patient visits, promote patient compliance, and improve the treatment outcomes. Thus, single administration of MINO incorporated in electrospun PLGA membranes for sustained release is a promising therapeutic strategy.
